# Prolonged persistence of a novel replication-defective HIV-1 variant in plasma of a patient on suppressive therapy

**DOI:** 10.1186/s12985-016-0617-0

**Published:** 2016-09-21

**Authors:** Samantha Rassler, Roberto Ramirez, Nadeen Khoury, Gail Skowron, Gautam K. Sahu

**Affiliations:** 1 HIV Biology and Persistence Laboratory, Division of Infectious Diseases, Roger Williams Medical Center, 825 Chalkstone Ave, Providence, 02908 RI USA; 2 Present address: University of Massachusetts-Boston campus, 100 Morrissey Blvd, Boston, 02125 MA USA; 3 Department of Internal Medicine, Yale School of Medicine, 330 Cedar St, New Haven, 06510 CT USA

**Keywords:** HIV-1, Antiretroviral therapy, Residual plasma viremia, 5′-major splice donor motif, Novel mutation

## Abstract

**Background:**

Cell-free residual HIV-1 virions (RVs) persist in plasma below 20–50 vRNA copies/ml in most patients on suppressive antiretroviral therapy (ART). How RVs are produced in the body during therapy is not fully clear. In this study, we have attempted to characterize these viruses of an ART-treated patient in vitro in order to gain insights into the mechanism of their production in vivo.

**Methods:**

We have reconstructed almost the entire genomes of RVs as DNA forms using the patient’s residual plasma vRNA by an overlapping RT-nested PCR method, and then sequence-analyzed the cloned genomes and tested them for their biological activities in vitro.

**Results:**

We found that the reconstructed molecular clones of RVs lacked antiretroviral drug-resistant mutations, as well as G-to-A hypermutations. The vDNA clones, when transfected into TZM-bl cells, released HIV-p24 into the culture media at extremely low levels. This low-level virus production was found to be due to the presence of a unique mutation (GU-to-GC) in the conserved 5′-major splice donor (MSD) motif of the corresponding vRNAs. We found that the incorporation of this point mutation by itself could cause defects in the replication of a standard HIV strain (JRCSF) in vitro. However, this novel viral variant was intermittently detected at 5 of 7 time-points in the patient’s plasma over a period of 39 months during therapy.

**Conclusions:**

This is the first identification of a natural point mutation (GU-to-GC) in the conserved 5′-MSD motif of HIV genomic RNA. The intermittent but prolonged detection of this replication-defective HIV variant in the patient′s plasma among other viral populations strongly suggests that this variant is released from highly stable productively infected cells present in vivo during therapy. The potential implication of this observation is that the elimination of such productively infected cells that contribute to residual viremia during suppressive therapy could be an important first step towards achieving a cure for HIV.

**Electronic supplementary material:**

The online version of this article (doi:10.1186/s12985-016-0617-0) contains supplementary material, which is available to authorized users.

## Background

Current ART suppresses plasma viral loads to levels below the limit of detection in clinical viral load assays, generally 20–50 HIV RNA copies per ml of plasma [1]. This suppressed viral load status remains stable as long as patients continue therapy with high adherence [2]. However, ultrasensitive laboratory-based techniques can detect very low amounts of vRNA circulating in plasma below the clinical detection limit in most patients on prolonged ART [3–9]. These low viral loads in plasma during therapy are referred to as residual viremia. The cell- or tissue source of the corresponding viruses (i.e., RVs) or their mechanism of production is still not fully clear.

A notion is that RVs are produced due to incomplete inhibition of viral replication by ART in the lymphatic tissues where antiviral concentrations appear suboptimal [10]. The process of continuous cycles of viral infection, integration, and production from target cells is referred to here as viral replication. However, viral variants resistant to antiretroviral drugs do not usually emerge in patients with high adherence to prolonged suppressive therapy [11–13]. Also, the levels of residual viremia were not affected by therapy intensification [14], even with the inclusion of an integrase inhibitor to the antiretroviral regimen [15–18]. Although some studies had observed transient increase in the levels of 2-LTR circles in a portion of patients who received raltegravir-based therapy intensification, and suggested the occurrence of viral replication at low levels during therapy [19, 20], others found no such increase in 2-LTR circles, contradicting the possibility of RV production as a result of ongoing replication during therapy [21]. Overall, these conflicting observations led to an alternative postulate that highly stable, productively infected cells may exist in lymphoid tissues or some bodily locations, where they persistently release virus particles during effective therapy [14, 22, 23]. Such reservoir was recently named as the active HIV reservoir [24], but direct evidence behind the postulate is still lacking.

To understand the mechanism of how RVs appear in plasma during therapy, we sought to molecularly clone these viruses, and then study their biology in vitro. Accordingly, we reconstructed eight RV clones from an ART-treated patient’s plasma vRNA by adapting a protocol published previously [25]. We found that the vDNA clones could secrete HIV-p24 in media upon transfection into a human cell line (TZM-bl), but the levels were much lower compared to the levels produced by the standard HIV_JRCSF_ clone [26], or by our previously published RV clone, C1P [25]. The presence of a unique mutation (GU-to-GC) in the conserved 5′-major splice donor (5′-MSD) motif of genomic vRNA was found to be a cause for diminished viral p24-production from RV clones. The known antiretroviral drug-resistant mutations as well as G-to-A hypermutations were absent in these clones. Interestingly, we could intermittently detect the unique viral 5′-MSD variant in the patient’s plasma over a period of 39 months during therapy, implicating that its source in the body remained highly stable and capable of producing virus at low levels during suppressive ART, contributing to residual viremia.

## Methods

### Study patient

**Fig. 1:**
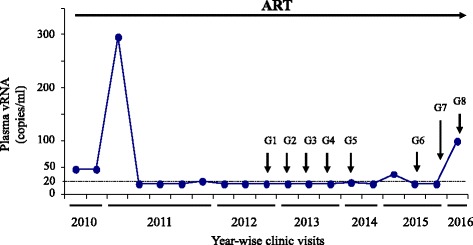
ART-mediated suppression of plasma viral load (VL) in patient G. Plasma VLs were measured by clinical assays with detection limits of 48 (prior to March 2011) or 20 copies/ml; results below the limit of detection were graphed as 47 and 19 copies/ml, respectively. Two VL blips of 297 and 100 copies/ml occurred in January 2011 and May 2016, respectively. Blood samples collected at 1st visit, 2nd visit, and so on in the study are indicated by G1, G2, etc., respectively

A white (non-Hispanic) 57-year-old male HIV+ patient (identified here as patient G) effectively treated with ART in the RWMC clinic was recruited in November 2012 for an HIV-1 reservoir study. He signed a consent form approved by the Institutional Review Board to participate in the study. During each visit to the study every 3 to 7 months, his plasma viral load was measured and he was asked to donate 50 ml of blood. Initially, for a period of 16 months, a total of five blood samples (G1 to G5 in Fig. 1) were collected from him, and about a year after the 5^th^ visit, three more blood samples (G6 to G8) were collected (Fig. 1), accumulating a total of 8 samples over the study period of 42 months.

### Blood sample processing

Each sample was immediately processed after collection through Ficoll-hypaque method as described previously [27]. Plasma virus particles were pelleted by ultracentrifugation, followed by vRNA isolation as previously performed [25, 28] using a vRNA isolation kit (Qiagen). The isolated plasma vRNAs were designated sequentially as G1P, G2P, etc., as they were isolated from blood samples collected at the 1^st^ visit (G1), 2^nd^ visit (G2), and so on, respectively (Fig. 1), and then stored at −70 °C for future use.

### Isolation and culture of CD4+ T-cells from blood

Whenever required, CD4+ T-cells were isolated from normal HIV-negative donors’ PBMCs through negative selection method using the EasySep human CD4+ T-cell enrichment kit (StemCell, Canada). The isolated cells were stimulated with Dynabeads human T-activator CD3/CD28 (ThermoFisher Scientific) by following the manufacturer’s instructions and cultured in media containing IL-2 (100U/ml) for expansion.

### RT-nested PCR, and cloning

**Fig. 2:**
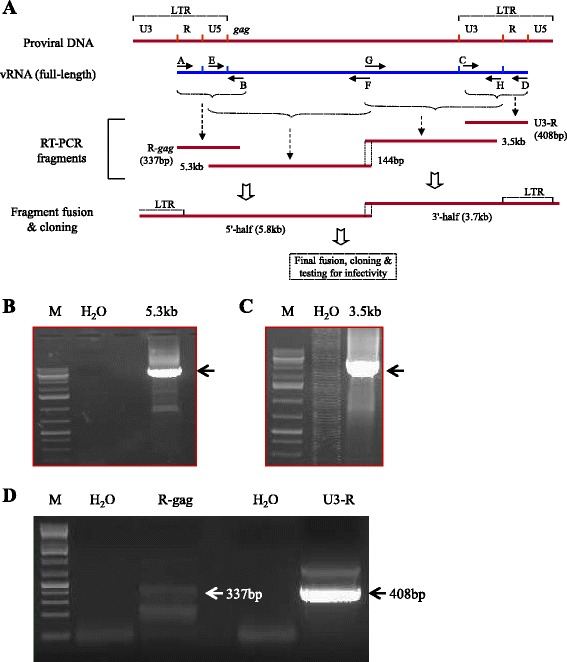
Reconstruction of residual plasma HIV-1 genomes as DNA form. Panel **a** schematically shows the corresponding strategy. The small 337 bp (R-gag) and 408 bp (U3-R) DNA fragments and the large 3.5 kb, and 5.3 kb fragments were separately amplified by RT-nested PCR using residual plasma vRNAs as targets. Each horizontal small arrow indicated by A through H represents a pair of primers (Additional file 5: Table S1) used in the RT-nested PCR. The U3, R and U5 regions are the elements of the HIV-1 long-terminal repeat (LTR). *R-gag* and *U3-R* fragments were fused to generate the *U3-gag* fragment (not shown), which was later combined with 5.3 kb and 3.5 kb fragments to build the 5′- and 3′-halves, respectively (see Additional file 1). Panels **b**, **c** and **d** show the amplified fragments (indicated by *arrows*) in agarose gels. Lanes are marked with the corresponding fragment names. Lane M shows the GeneRuler 1 kb plus DNA ladder (Thermo Scientific). In panel **d**, the size difference between the R-gag and U3-R bands is not reflected in the agarose gel due to anomaly in the migration of DNA bands, but the identity of these bands was verified by sequence analyses. A 200 bp band seen below the 337 bp *R-gag* band is not HIV-specific

Four different fragments spanning almost the entire HIV genome (Fig. 2a) were amplified by RT-nested PCR, as previously described [25] and the amplified fragments are shown in Fig. 2, panels b-d. Twenty-one microliters of vRNA equivalent to about one-sixth of the total vRNA isolated from 50ml blood-derived plasma were used as the template in the first RT-PCR step. For such low copy number target vRNA amplification, a highly sensitive and efficient one-step RT-PCR system that includes SuperScript III/platinum Taq (Life Technologies) was employed using the appropriate primer pairs and reaction conditions (see Additional file 1). After 40 cycles of PCR, 5 μl of the products were taken as templates in a 50 μl reaction volume for nested PCR of 35 cycles using the Expand high-fidelity PCR system (Roche). The amplified products were eluted from 0.8 % agarose gel and cloned into pCR-XL-TOPO vector (Life Technologies) in TOP10 competent cells for sequencing and analyses.

An overlapping PCR method was performed as previously described [25] to attach two different amplified fragments as appropriate using the Expand high-fidelity PCR system (Roche) but after removing any 3′-A overhangs of amplified fragments with T4 DNA polymerase. The resulting fragments were cloned into pCR-XL-TOPO vector and sequenced to validate the correct fusion of fragments.

### Sequence analyses

Nucleotide (nt) sequences were aligned using the CLUSTAL-W program available in Vector-NTI express software (Life Technologies), BioEdit or, in MEGA6 program [29]. The quality of the full-length viral genomic sequences was checked by using the QC tool available in the HIV database of Los Alamos National Laboratory.

### Phylogenetic analyses

Residual viral sequences were subjected to phylogenetic tree reconstruction using the Neighbor-Joining method [30] in the MEGA6 program [29]. The evolutionary distances were computed using the Tamura-Nei method [31] and the rate variation among sites was modeled with a gamma distribution (shape parameter =1). The corresponding sequence regions from the standard laboratory HIV strains, such as HIV_NL4–3_ [32], HIV_213_ [33], HIV_JRCSF_ [26], HIV_C1P_ [25], were included in the outgroup. The bootstrap test (1000 replicates) was performed [34] and the values are shown next to the branches.

### Transfection of plasmid DNA into TZM-bl cells

These cells [35] were cultured in Dulbecco’s Modified Eagle Medium (DMEM) supplemented with 10 % fetal bovine serum (FBS), 2 mM L-glutamine and penicillin/streptomycin. One microgram of plasmid DNA was transfected into TZM-bl cells in 12-well plate using lipofectamine (Life Technologies) DNA transfection reagent by following the manufacturer’s instructions. The cultures were incubated in a 5 % CO_2_ incubator at 37 °C. The next day, media from the transfected cells was replaced with fresh media, and the cultures were continued for another 24-48 h.

### Site-directed mutagenesis

This was performed by using the GeneArt Site-directed Mutagenesis Kit (Life Technologies). Primers were designed by following the GeneArt designer tool (Life Technologies), and after every mutagenesis reaction, the desired change in DNA was confirmed by sequencing.

### Sequence deposition into the GenBank

Nucleotide sequences for the reconstructed residual plasma HIV-1 clones have been submitted to GenBank under accession numbers KT284371 to KT284378.

## Results

### Status of clinical viral loads during prolonged ART and longitudinal sampling of residual plasma vRNA

Patient G was 57 years old in 2012 when he donated his first blood sample for this study. Based on the available health records, he started ART in 1991 and had been effectively treated for HIV infection in the RWMC clinic. He was treated with the antiretroviral combination, tenofovir/emtricitabine and nevirapine during the first year of the study, and then with the current combination of abacavir sulfate/lamivudine and nevirapine. From April 2010 to May 2016, his plasma viral loads remained <50 HIV RNA copies/ml in clinical tests performed every 2–7 months during his clinic visits, except that he had two viral load blips of 297 and 100 copies/ml in January 2011 and May 2016, respectively (Fig. 1). Clinical viral load test results were below the 20 copies/ml limit of detection in approximatley 75 % of his visits during the last 5 years, indicating that viremia was effectively suppressed by the prescribed antiretroviral regimen. During this period, his CD4+ T-cell counts/μl of blood ranged from 608 to 1106.

### Reconstruction of the full-length residual HIV genomes

The underlying strategy for this reconstruction was primarily adapted from our previously published report [25] and is schematically shown in Fig. 2a. Essentially, four viral genomic fragments were amplified (Fig. 2, panels b-d) from G4P vRNA by RT-nested PCR (see methods). Each of the amplified 5.3 kb, 3.5 kb, 337 bp and 408 bp fragments (Fig. 2a) had an overlapping region(s) with its neighboring fragment(s). The overlapping regions were exploited to combine the neighboring fragments either by the overlapping PCR method or by the restriction enzyme digestion and ligation method as appropriate in order to reconstruct the full-length vDNA genomes. For instance, 337 bp *R-gag* and 408 bp *U3-R* fragments were combined through the overlapping PCR method to generate the *U3-gag* fragments, which were also subsequently combined with the 5.3 kb and the 3.5 kb fragments through the same method to build 5′- and 3′-halves of the viral genome. We took two different 5′-halves and combined each with 4 different 3′-halves (Additional file 2: Figure S1) by utilizing the overlapping EcoN1 sites and generated a total of 8 full-length residual vDNA clones (namely, RV-1, −1a, −1b, −1c, −11a, −11b, −11c, and -11d).

**Fig. 3:**
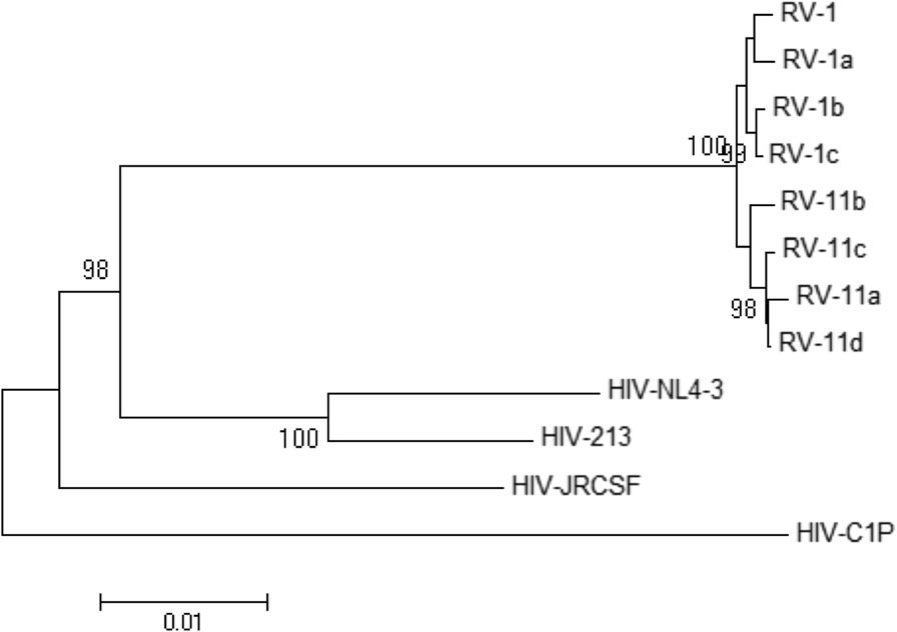
Phylogenetic analyses of RV sequences derived from eight reconstructed clones. Viral sequences (~9.6 kb each) were subjected to phylogenetic tree reconstruction by using the Neighbor-Joining method. Bootstrap values greater than 70 % are shown. HIV-NL4-3, HIV-213, HIV-JRCSF and HIV-C1P represent the standard HIV strains used in the laboratory

The nt sequences of these viral clones were deposited into GenBank (see methods) and were identified as the HIV subtype B sequences by using the NCBI HIV subtyping tool (http://www.ncbi.nlm.nih.gov/projects/genotyping/formpage.cgi). These viral nt sequences were unique showing maximum homologies up to 92-94 % with the existing HIV sequences in the NCBI nt database. The phylogenetic tree (Fig. 3) constructed by using the Neighbor-joining method [30] showed that the RV sequences clustered together in a branch separated from those of laboratory standard HIV sequences, suggesting that the obtained viral sequences were patient-specific, not laboratory contaminants.

### The reconstructed full-length residual vDNAs could secrete HIV-p24 in media at low levels upon transfection into TZM-bl cells

**Fig. 4:**
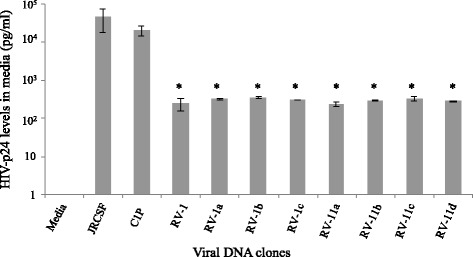
Limited levels of HIV-p24 production from all residual vDNA clones. TZM-bl cells were transfected separately with eight reconstructed vDNA clones in duplicate, and after 48 h, HIV-p24 levels in culture media were quantified by ELISA (Advanced Bioscience, Inc.). A standard HIV (JRCSF) DNA and our previously published residual vDNA clone (C1P) were used as positive controls for virus production in these assays. Error bars represent standard deviations (s.d.). Results were compared with the positive control C1P using unpaired *t*-test; asterisk (*) indicates *p* < 0.05

To examine whether the viral genomic clones are capable of producing virus in vitro, we transfected vDNAs into TZM-bl cells in 12-well plate (see methods). For positive controls, equivalent amounts of the standard HIV_JRCSF_ DNA and the previously cloned residual HIV_C1P_ DNA were also transfected into TZM-bl cells separately. After 48h of incubation, culture supernatants were tested for the levels of HIV-p24 by enzyme-linked immunosorbent assay (ELISA). Figure 4 shows that all residual vDNA clones reconstructed in this study could produce HIV-p24 in transfected cells, which was secreted into the culture media. However, we repeatedly observed that the levels of secreted HIV-p24 were ~2-log_10_ lower than the levels detected with either of the positive controls, i.e., HIV_JRCSF_ or HIV_C1P_ DNA (Fig. 4). Although culture media from vDNA-transfected cells showed marginal levels of infectivity in MAGI assays [36], the released virions in media were defective for replication in activated CD4+ T-cells or monocyte-derived macrophages of normal donors (data not shown).

### Residual viral sequences lacked G-to-A hypermutation as well as drug-resistant mutation

To investigate the underlying reason for the limited viral production as well as the replication-defect of the virions produced from the cloned vDNAs, we sought to examine whether these clones possess G-to-A hypermutations at statistically significant levels compared with the standard HIV_NL4–3_ clone. G-to-A hypermutations (or transitions) occur in proviral sequences due to the mutational pressure exerted by the APOBEC-induced cytosine deamination on nascent viral cDNA during reverse-transcription in the infected cells [37–39]. An excess of such mutations, including premature stop codon generation in the viral ORFs [40, 41], results in viral replication defects. Although the viral Vif counters the negative effects of APOBEC proteins on the virus in host cells [42], G-to-A hypermutations still occur in patients’ PBMC-associated proviral sequences [43]. Upon analyses of the full-length residual vDNA sequences using the Hypermut 2.0 program [44] available in the Los Alamos National Laboratory (LANL) website, we found that none of the eight clones had G-to-A hypermutations when compared with the standard HIV_NL4–3_ sequence (Fisher’s exact P-values range from 0.91 to 0.95, where *P* < 0.05 would indicate hypermutation). These data indicate that the cloned RVs were devoid of hypermutations; this observation is in agreement with a previously published report [40]. By using the QC tool available in the LANL, we found that all the viral open reading frames (ORFs) in these full-length clones were also intact. Overall, these data suggest that the detrimental mutations did not accumulate to account for HIV clones’ low viral productivity or replication defects in vitro.

Since the vDNA clones were derived from vRNA isolated from plasma during suppressive therapy, an obvious question arises whether the cloned RVs possessed ART-resistant mutations for which they were able to somehow replicate in vivo at low levels during therapy. We found none of the viral *pol* ORFs possessed any of the drug-resistant mutations listed in the Stanford University’s HIV Drug Resistance Database, suggesting that the cloned RVs represent drug-sensitive viruses circulating in plasma during therapy.

### A novel mutation identified in the conserved 5′-MSD site of vRNA caused limited HIV-p24 production from residual vDNA clones

**Fig. 5:**
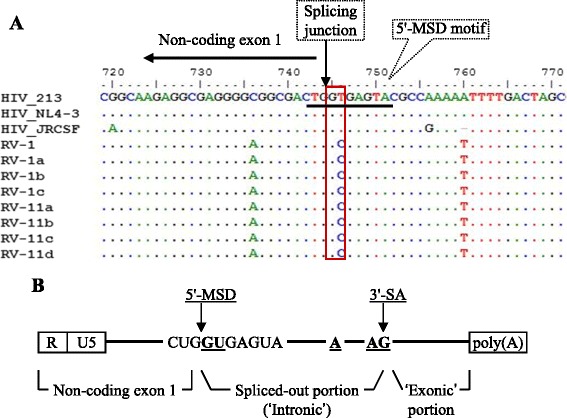
Analyses of viral genomic region containing 5′-MSD motif. **a** The presence of a unique GT-to-GC mutation at the 5′-MSD motifs of residual vDNA clones. Only a region spanning the 5′-MSD junction was taken from each vDNA sequences and aligned with the corresponding sections of the three standard HIV strains (top). The 5′-MSD motif mutation (GT-to-GC) in each vDNA clone is shown within the vertical box. **b** Illustration of the conserved splicing motifs present in HIV genomic RNA. Bold underlined GU, A and AG represent 5′-major splice donor (5′-MSD) site, branch-point, and a 3′-splice acceptor sites (3′-SA), respectively. Generally, such sites are also utilized during mRNA splicing in eukaryotes. The R and U5 regions belong to the HIV long-terminal repeat (LTR). The ‘non-coding exon 1’ is always combined with the polyadenylated [poly(A)] ‘exonic’ portion of vRNA upon splicing

We sought to compare residual vDNA sequences with those of three standard strains (HIV_213_, HIV_NL4–3_ and HIV_JRCSF_) in the CLUSTAL-W program [45] to identify any unique mutations present in the residual vDNA clones, which could potentially be associated with the low p24-production phenotypes of these clones. Among many sequence changes, strikingly, we observed a single nt mutation (GT-to-GC) present in the 5′-MSD motif of each RV clone (Fig. 5a), which was not previously reported for HIV in nature. However, the artificial mutation of GT-to-CA at the 5′-MSD motif of the standard HIV_NL4–3_ clone was previously shown to cause lower efficiencies of vRNA splicing in the transfected HeLa cells, resulting in ~6-fold lower levels of viral progeny formation compared to the levels observed with the wild-type virus [46]. The importance of the 5′-MSD motif in HIV-1 biology is briefly mentioned in the discussion section, but the conserved motifs for splicing are illustrated in Fig. 5b.

**Fig. 6:**
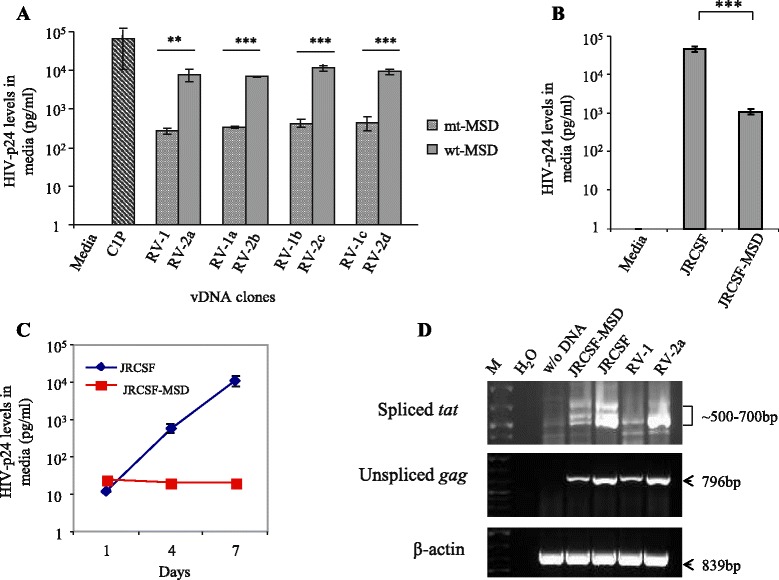
Increased HIV-p24 production from vDNA clones with wild-type 5′-MSD motif. **a** The GC-to-GT reversion at the 5′-MSD motif of residual vDNA clones improved viral p24 production. Equivalent amounts of vDNAs were transfected into TZM-bl cells, and 48h later, HIV-p24 levels in culture media were quantified by ELISA. C1P, a standard residual HIV-1 clone, was used as a positive control. mt-MSD and wt-MSD represent RV clones with mutated 5′-MSD and their wild-type counterparts, respectively. Error bars represent s.d., *n* = 4. The statistical significance of the data was calculated by using unpaired *t*-test; asterisk (**) and (***) indicate *p* < 0.002 and *p* < 0.0001, respectively. **b** Reduced HIV-p24 production from the standard HIV-JRCSF clone with 5′-MSD motif mutation. GT-to-GC mutation was incorporated at the 5′-MSD motif of HIV-JRCSF clone by site-directed mutagenesis. Equal amounts of the wild-type (JRCSF) and the mutant (JRCSF-MSD) vDNA clones were transfected into TZM-bl cells in triplicate, and 48h later, the levels of p24 production in culture media were measured by ELISA. Error bars represent s.d. The statistical significance of the data was calculated using unpaired *t*-test. Asterisks (***) denote *p* < 0.0005. **c** Replication defect of JRCSF-MSD mutant in stimulated CD4+ T-cells. HIV-JRCSF and JRCSF-MSD viruses were freshly prepared by transfecting their DNA clones into TZM-bl cells, followed by harvesting culture media after 48h. About 1x10^6^ of freshly stimulated CD4+ T-cells from a normal donor were infected overnight in triplicate in 24-well plate with the equivalent amounts of these viruses (1200 pg of p24 each). Next day, infected cells were washed three times and cultured in media containing 100 u/ml of IL-2. Culture supernatants were harvested on days indicated and tested for the levels of HIV-p24 by ELISA. Error bars represent s.d. **d** Low-levels of spliced vRNA produced by 5′-MSD mutated vDNAs. Total RNA isolated from TZM-bl cells transfected equivalently with various vDNA clones were used as template for RT-PCR amplification of HIV transcripts. RV-1 and RV-2a represent residual HIV clones with mutated and wild-type MSD motifs, respectively. Panels are labeled to the left with the corresponding amplified products. Lane M denotes GeneRuler plus DNA ladder

To examine whether the natural GT-to-GC mutation at the 5′-MSD site of our cloned vDNAs caused limited HIV-p24 production in transfected cells, we reverted the GC dinucleotide back to the wild-type GT form at the 5′-MSD sites of four vDNA clones (i.e., RV-1, −1a, −1b, and -1c) by site-directed mutagenesis (see methods). The resulting vDNA clones were labeled as RV-2a, −2b, −2c and -2d (Fig. 6a). Upon transfection into TZM-bl cells, we found that these new vDNA clones (RV-2a to RV-2d) with the wild-type 5′-MSD motif could produce on average ~20 to 30-fold higher levels of HIV-p24 than their respective parental clones with mutated 5′-MSD motif (Fig. 6a), suggesting that the 5′-MSD motif mutation did play a significant role in the limited production of HIV-p24 from the original vDNA clones.

To further substantiate the above observation, we incorporated GT-to-GC mutation at the 5′-MSD motif of the standard HIV_JRCSF_ clone by site-directed mutagenesis; the levels of p24 production from the resulting mutant clone in transfected cells were then compared with those of the wild-type counterpart. We found that the 5′-MSD mutated HIV_JRCSF_ clone could produce ~42-fold lower levels of HIV-p24 than the wild-type clone (Fig. 6b); however, the mutant virus could not replicate in activated CD4+ T-cells in vitro (Fig. 6c). These data demonstrated that the 5′-MSD motif mutation in the original reconstructed vDNA clones was responsible for reduced HIV-p24 production in the transfected TZM-bl cells, and the mutation by itself could impede viral replication in activated CD4+ T-cells in vitro.

As mentioned above, the GT-to-CA mutation (two-nt change) at the 5′-MSD motif of HIV_NL4–3_ was previously reported to cause low efficiencies of vRNA splicing in HeLa cells [46], and also, the mutant virus was found to utilize a cryptic splice donor GT (GU for RNA) site located 4 nt downstream of the original 5′-MSD GU motif for vRNA splicing (see Fig. 5b). To examine whether the single nucleotide change (GT-to GC) in the 5′-MSD motif of the cloned RVs and HIV-JRCSF can affect vRNA splicing, we transfected a RV 5′-MSD mutant (RV-1) clone and its wild-type counterpart (RV-2a), as well as JRCSF and JRCSF-MSD clones, into TZM-bl cells separately (see Additional file 1). Forty-eight hours later, we found that RV-1- and JRCSF-MSD-transfected cells showed much reduced levels of spliced viral *tat* mRNA, compared to RV-2a and JRCSF-transfected cells (Fig. 6d), respectively. Furthermore, the viral mutants could utilize the GU site located 4nt downstream of the original 5′-MSD GU site for low-level vRNA splicing (Additional file 3: Figure S2), as similar to the NL4-3 mutant [46]. These data suggest that the single nucleotide mutation (GT-to GC) at the 5′-MSD motif of HIV can also lower vRNA splicing efficiencies in vitro, and provide an explanation for why limited levels of HIV-p24 are produced by 5′-MSD mutated vDNAs, in comparison to their wild-type counterparts, in transfected cells (Fig. 6a and b).

### Viral 5′-MSD mutant was intermittently detected in the patient’s plasma for a prolonged period during therapy

The isolation of replication-defective RV 5′-MSD mutant from the ART-treated patient’s plasma at his 4^th^ visit led to a possibility that some productively infected cells persisting in the body were able to express the defective virions and release them into plasma, even in the presence of therapy. Such infected cells releasing virus during therapy were previously predicted to exist in vivo [14, 22, 23] and recently termed as the active reservoir of HIV [24], which was also thought to remain highly stable in the body and contribute to residual viremia during therapy. If 5′-MSD variant was released into plasma from such a stable virus-expressing reservoir, one would expect to detect the same 5′-MSD variant in plasma at different time points over a prolonged period. To examine this possibility, we amplified the *R-gag* fragments containing the 5′-MSD motif from other longitudinally-collected plasma vRNA samples (i.e., G1P-, G2P-, G5P- to G8P-vRNAs) by RT-nested PCR (Additional file 4: Figure S3) and sequence-analyzed them. We found that the 5′-MSD variant could be detected in 5 out of 7 plasma samples analyzed (Table 1); the interval between the first and the last detection was about 39 months (Table 1). The viruses with wild-type 5′-MSD motif were also detected along with the variant in most (6/7) samples (data not shown in Table 1). However, the prolonged but intermittent detection of the replication-defective viral 5′-MSD variant in plasma strongly suggests that at least this variant was released from a cellular source that remained extremely stable in the body during therapy.

**Table 1:** Prolonged detection of viral 5′-MSD variant in plasma during therapy

Plasma vRNA (sample ID)	G1P	G2P	G3P	G4P	G5P	G6P	G7P	G8P
Collection date	Nov. 13, 2012	Feb. 12, 2013	July 23, 2013	Oct. 28, 2013	Mar. 11, 2014	Apr. 21, 2015	Nov. 24, 2015	May 20, 2016
Detection of RV 5′-MSD variant	no	yes	nd	yes	no	yes	yes	yes
Days since first detection	−91	0		258		798	1015	1193

‘nd’ indicates ‘not done’

## Discussion

Residual plasma viremia persists in most patients below the clinical detection limit, even after prolonged suppressive therapy [3–9]. The clinical significance and the cellular source of this low-level viremia are still not fully clear. HIV is known to persist in various cellular or tissue reservoirs in ART-treated patients, from where it can disseminate infection in the body when the therapy is stopped, resulting in viral load rebound in plasma within a few weeks [47]. By virtue of viral expression status, HIV reservoirs can be classified into two categories: one as the ‘latent reservoir’ [48], and the other as the ‘active reservoir’ [24]. The latent reservoir classically consists of resting memory CD4+ T-cells carrying proviral DNA in a dormant state. While this reservoir can produce virions only after cell-activation [49], previous studies had undermined its contribution to persistent residual viremia during therapy upon analyzing patients’ blood samples, and proposed the existence of an unknown source in vivo for such low-level viremia [28, 50]. Recently, it was hypothesized that this unknown reservoir (namely, the active reservoir) remains highly stable in vivo and persistently produces virions at low levels during therapy, as opposed to the latent reservoir [24]. Viral production is referred to here not as viral replication that occurs through complete cycles of infection, integration and production of progeny virions; but rather simply as spontaneous viral synthesis and release from already infected cells, even in the presence of therapy. The existence of such a reservoir in patients is generally difficult to prove, especially in light of a recent report by Lorenzo-Redondo, et al. [13] describing that viral replication continues in tissues where antiretroviral drug concentrations are suboptimal. The authors also proposed that under low ART-pressure, the evolution of drug resistance is greatly diminished by replication fitness advantages of wild-type viruses over drug-resistant variants colocalized in the tissue compartments, providing an explanation for why drug-resistant viral variants do not usually emerge in patients on prolonged suppressive therapy.

The residual vDNA clones that we have reconstructed from the patient’s plasma vRNA isolated at G4 (Fig. 1) did not possess any antiretroviral drug-resistant mutation or G-to-A hypermutations, and had all viral ORFs intact. However, the virions released from vDNA-transfected cells could not replicate in activated CD4+ T-cells or monocyte-derived macrophages of normal donors (data not shown). The absence of viral replication in vitro indicates that the reconstructed vDNAs represented replication-defective cell-free RVs that were circulating in patient G’s blood during his 4^th^ visit to the study. Upon investigation, we found that all vDNA clones had a unique GU-to-GC mutation at the 5′-MSD motifs of the corresponding viral genomic RNAs, and the reversion of this mutation to wild-type led to significantly increased levels of HIV-p24 production in the transfected TZM-bl cells, validating that this mutation limits HIV expression (Fig. 6a).

The 5′-MSD motif is one of the four splicing donor (SD) sites in the HIV-1 RNA genome, which remains highly conserved and is constitutively used during the processing of full-length vRNA through splicing to generate over 40 different viral mRNA species [46, 51–53]. There are about 8–11 3′-splice acceptor (SA) sites in the viral genome [52, 53]. In the RNA splicing mechanisms, the cellular spliceosome cleaves at exon-intron junctions through trans-esterification reactions [54–56], typically right before the GU dinucleotide (underlined) at the 5′-MSD site (CUGGUGAGUA), and right after the AG dinucleotide at the 3′-SA sites in vRNA to remove intronic portion (see Fig. 5b). The noncoding exon 1 spanning upstream of the 5′-MSD site is always included with all spliced forms of vRNA [53]. Generally, the GU dinucleotide motif at the mRNA splice donor site is well conserved across the species and possessed by ~98 % of introns [57, 58], but the mutations at this site are known to affect mRNA splicing negatively in vitro [59, 60].

The 5′-MSD mutation (GT-to-GC), to our knowledge, has not been previously reported for HIV in the natural context. While we could detect this mutation in patient G’s plasma frequently (5 of 7 occasions), we failed to detect the same mutation in residual plasma vRNAs of three other patients (not shown) on suppressive therapy, suggesting that the 5′-MSD mutation in HIV infection may occur rarely. The introduction of the same 5′-MSD mutation (i.e., GT-to-GC) in the standard HIV strain (JRCSF) was found to diminish viral RNA splicing (Fig. 6d) and gene expression severely (Fig. 6b), and the viral mutant (JRCSF-MSD) could not replicate in activated CD4+ T-cells of normal donors (Fig. 6c), which strongly suggests that the RV 5′-MSD variant remains replication-defective in vivo. Therefore, the 5′-MSD mutation should not offer the virus a selective advantage in vivo, except that the down-modulation of viral gene expression caused by the mutation may facilitate the host cells to survive in vivo by avoiding viral cytopathic effects as well as host immune clearance mechanisms. Interestingly, the defective variant was detected in the patient’s plasma at 5 out of 7 time-points that spanned over 42 months during the study (Table 1), suggesting that its cellular source persisted long-term in the body during suppressive therapy and released virions in plasma at low levels, contributing to residual viremia.

The low-level production and thereby shedding of 5′-MSD variant into plasma from a source cannot be confused with ‘cryptic’ viral replication (which is suspected to occur in tissues during therapy [13]), because the 5′-MSD mutation by itself could impede viral replication, even if other viral components remained intact (Fig. 6c). Instead, the long-term detection of replication-defective 5′-MSD mutant in plasma during suppressive therapy should reveal that some highly stable infected cells existing in the body are able to express virions at low levels at the single cell-level and release them into plasma, even in the face of therapy without experiencing viral replication events at the cell-population levels. As mentioned earlier, such stable HIV-infected cells releasing virus were previously predicted to exist in vivo during therapy [14, 22–24]; however, direct evidence to support this hypothesis was largely lacking. We believe that our data demonstrating the prolonged persistence of the replication-defective 5′-MSD variant in the patient’s plasma during therapy serves as evidence for the existence of stable HIV-producing cells (namely, the active reservoir) in vivo. It is worth noting that we could also detect viruses with wild-type 5′-MSD motif in all plasma samples of the patient, except in one, which indicates that residual plasma viruses may not be all genetically defective but could be a mixture of particles carrying replication-defective and -competent genomic RNAs, as previously reported [25].

**Fig. 7:**
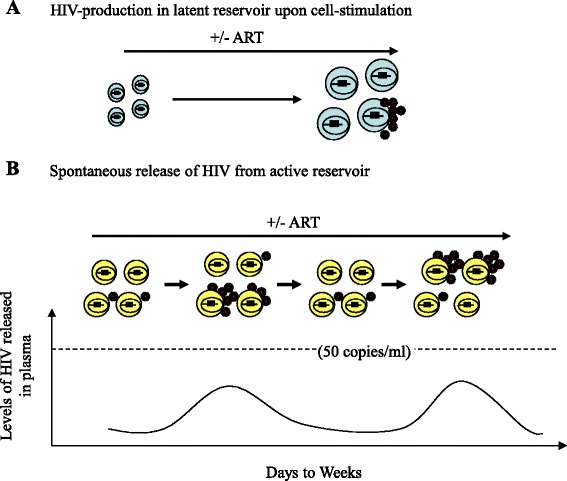
Status of HIV expression in different reservoirs. **a** Latent reservoir. HIV is not usually expressed, but upon cell-stimulation, only a fraction of inducible latent proviruses residing in resting memory CD4+ T-cells (shown in *blue*) can be reactivated to express virus in the presence or absence of ART. **b** Active reservoir. A model for RV-production from a pool of infected ‘semi-quiescent’ cells (shown in *yellow*) representing an expanded cell clone is illustrated here. Virus production may occur spontaneously in waves over time with or without ART. Such multiple clonally expanded infected cell lineages in tissues may simultaneously release virions during therapy, each contributing to residual viremia

Although we cannot rule out the possibility that residual viremia may partly emanate from ‘cryptic’ viral replication occurring in tissues during therapy [13], the replication fitness advantage model proposed by these authors does not fit with the appearance of replication-defective 5′-MSD variant in the patient’s plasma in our study. How this viral variant was produced is not clear, but the underlying mechanisms may shed light on the very nature of the source of residual viremia persisting during therapy. Based on the intermittent detection of 5′-MSD variant in plasma below the clinical detection limit, as well as the published data by others [61], we propose a model (Fig. 7b) illustrating how RVs might be produced from a source in vivo. It is known that the current ART does not block virion-production or -release from the cells that are already infected and remain in the productive phase of infection. A stable pool of infected cells residing in tissues at low frequencies may actively release virions at low levels over time, giving rise to residual viremia during therapy. While these cells may not usually circulate in blood [28, 50], they could be clonally expanded in tissues, like latently infected CD4+ T-cells [62, 63], to form cell lineages carrying genetically distinct but phylogenetically related proviral species [64]. Recently, the detailed analyses of a highly expanded CD4+ T-cell clone carrying infectious proviral DNA (namely, AMBI-1) at the same integration site was reported [65]. This clone was identified in an HIV-infected patient who had squamous cell carcinoma. Although the clonal cells were detected in blood, as well as in lymphoid tissues, they were found enriched in metastatic lesion. Importantly, the AMBI-1 virus was persistently detected among residual virus populations circulating in plasma for more than 3 years, suggesting that the virus was released into plasma by this expanded clone for such a prolonged period [65]. We believe each of such highly stable infected cell lineages present in the body can release virions at low levels in waves over time (Fig. 7b), as observed by others in gut-associated lymphoid tissues of humanized mouse model of HIV infection [61], contributing to residual viremia during therapy. The activation state of these cells may remain confined in-between the resting and the fully activated states, allowing the virus to be expressed at low levels. The generation of such ‘semi-quiescent’ CD4+ T-cells persistently producing HIV at low levels for a prolonged period was previously observed in our H80 model of HIV latency [27]. It is not clear whether such low-level virus-expressing cells usually die in vivo, as proposed recently [66], or whether they persist as highly stable chronic virus producers during therapy. In our study, the reservoir expressing RV 5′-MSD variant is found to live in the patient for at least 39 months (Table 1), which suggests that such virus-expressing reservoir remains extremely stable in vivo. If the majority of RVs in treated patients’ plasma are released from stable HIV-producing cells (i.e., active reservoir) as similar to the 5′-MSD variant in our study, then therapy intensifications should not have any effect on the levels of residual viremia, as observed previously [14–18]. In contrast, the latent reservoir that consists of long-lived resting memory CD4+ T-cells carrying dormant but replication-competent proviral DNA requires cell-stimulation for virus production [49] (Fig. 7a). However, the majority of these proviral DNAs remain refractory to even optimal cell-activation in vitro [67].

A limitation of our study is that the cloned RVs could not replicate in stimulated blood CD4+ T-cells of normal donors, unlike in our previous study [25], even when the mutated 5′-MSD motif was reversed back to the wild-type form. We believe that the RV clones possess additional defect(s), other than the 5′-MSD mutation, which may have been incorporated due to PCR errors. Another limitation is that the cloned vDNAs might not represent the genetic materials of the natural residual virions present in plasma during sampling because of inherent imprecision of our multifragment amplification-based cloning strategy. Even so, the characterization of cloned vDNAs led us to identify the unique replication-defective viral 5′-MSD mutation and accumulate evidence for residual virion-synthesis and -release from highly stable productively infected cells present in vivo. Such stable productively infected cells constituting active reservoir may also release replication-competent RVs capable of spreading infection in vivo when therapy is interrupted [25, 28]. Other limitations of the study are that we have cloned and characterized RVs from only one patient on ART, and also we failed to detect the novel 5′-MSD mutation in RVs of another three treated patients (mentioned above). Studies on additional patients are required to further verify the RVs’ characteristics and the mechanism of their persistence in vivo. Nevertheless, the prolonged detection of the defective 5′-MSD variant in plasma of a single patient in this study, as well as the absence of drug-resistant mutations in the virions provide, for the first time, a strong molecular evidence for their release from a highly stable active reservoir present in his body during therapy. It would be interesting to examine whether any secondary lymphoid tissues, such as lymph node or rectal tissue, or others, in patient G possess HIV with 5′-MSD mutation to pose as a source of RVs in plasma during suppressive ART.

## Conclusions

Our data, although obtained from a single patient, indicate that highly stable HIV-producing cells can exist in vivo, even in the presence of suppressive therapy. These infected cells may pose as the previously hypothesized active reservoir, which can release virions into plasma either persistently or intermittently, contributing to residual plasma viremia during therapy. Should the active reservoir harbor and release replication-competent RVs in vivo during therapy, such viral source is expected to spread infection quickly after therapy interruption, giving rise to viral load rebound. Thus, the elimination of the active reservoir through new therapeutic strategies should remain urgent in the effort of HIV-1 eradication.

## Additional files

Additional file 1: Supplementary methods. (DOC 36 kb) Additional file 2: Figure S1. Combinatorial strategy to build 8 residual vDNA clones. Each 5′-half clones (i.e., clone 1 and 6 shown on the left) were combined with four different 3′-half clones on the right through the overlapping EcoN1 sites (**↓**). An additional EcoN1 site (׀) present in all 3′-half clones, except clone 1 (see right), was abolished by site-directed mutagenesis prior to the combination. (PPT 55 kb) Additional file 3: Figure S2. Shift of 5′-major splice donor cleavage site toward 4nt downstream in short transcripts of HIV 5′-MSD mutants. Panel **a**, **b**, **c**, & **d** represent the group of aligned sequences derived from viral short transcripts. Top sequences in each panel represent the vDNA clones from where the transcripts were synthesized and subsequently spliced in transfected cells. HIV-JRCSF-mt and RV-1 in panel **b** and **c** represent the sequences from 5′-MSD motif mutants of HIV-JRCSF and a reconstructed RV clone, respectively; whereas RV-2a in panel **d** indicate the sequences of RV-1, except that the dinucleotide ‘GC’ was reverted back to the wild-type ‘GT’ form at the 5′-MSD site (underlined in red). In Panel **b**, one of the 5 transcripts analyzed (i.e., clone 4) showed a new splicing cleavage point located 50nt downstream of the regular 5′-MSD cleavage site. In Panel **c**, a viral transcript represented by clone 2 utilized the dinucleotide GC at the mutated 5′-MSD motif as a non-canonical splice donor site for RNA cleavage. (PPT 95 kb) Additional file 4: Figure S3. Amplification of *R-gag* fragments by RT-nested PCR. Longitudinally-collected vRNA samples (i.e., G1P, G2P, etc.) were used as targets for amplification. (PPT 105 kb) Additional file 5: Table S1. Primers used in the RT-nested PCR for various fragment amplifications. (DOC 30 kb)

## Abbreviations

APOBEC: Apolipoprotein B mRNA-editing enzyme catalytic polypeptide-like
ART: Antiretroviral therapy
CD4: Cluster of differentiation 4
DNA: Deoxyribonucleic acid
HIV-1: Human Immunodeficiency Virus-1
MSD: Major splice donor
NCBI: National Center for Biotechnology Information
NJ: Neighbor-Joining
ORF: Open reading frame
PCR: Polymerase chain reaction
RNA: Ribonucleic acid
RT: Reverse transcriptase
RV: Residual HIV-1 virion
RWMC: Roger Williams Medical Center
