# Supplementary material for: Prolonged persistence of a novel replication-defective HIV-1 variant in plasma of a patient on suppressive therapy
Source: Virol J. 2016 Sep 21;13:157. doi: 10.1186/s12985-016-0617-0 (PMC5031319; doi:10.1186/s12985-016-0617-0)
Supplement: Additional file 1: — Supplementary methods. (DOC 36 kb) [file 12985_2016_617_MOESM1_ESM.doc]

**Supplemental Methods**

**RT-nested PCR reaction conditions**

Figure 2A schematically shows the amplified fragments spanning almost the entire HIV genome. Table S1 lists the primers used for these fragment-amplifications. For all 1^st^ step RT-PCR reactions, 50µl of the reaction mixture consisted of 0.3µM of each primer, 1x reaction buffer, and 2µl of superscript III RT/platinum Taq (a highly sensitive and efficient enzyme mixture purchased from Life Technologies). For the nesting steps, 5µl of products obtained in the RT-PCR were taken as templates in a 50µl reaction volume, giving rise to a 10-fold dilution of the products. The Expand high-fidelity PCR system (Roche) was used in these reactions.

For 3.5kb fragment amplification, 21µl of residual plasma vRNA, which were about 1/6 of the total vRNA isolated from 50ml of blood-derived plasma, was used as templates. cDNA synthesis was performed in one cycle at 50°C for 30 min, followed by 1 cycle of denaturation at 94°C for 2 min. Then 40 cycles of PCR amplification was carried out as follows: (denaturation) 94°C for 15 sec, (annealing) 57°C for 30 sec, (extension) 68°C for 6 min, and final extension of 1 cycle at 68°C for 10 min. For the nesting step, the reaction condition and cycles were similar to those of the RT-PCR step, except that only 35 cycles of amplification were carried out.

Reaction conditions for 5.3kb fragment amplification were similar as those of 3.5kb fragment amplification (above), except that the annealing temperature was 60°C for both steps of PCR.

For the amplification of U3-R and *R-gag* fragments, 15µl of vRNA were used as templates in the RT-PCR step. cDNA synthesis was performed in one cycle at 45°C for 15min, followed by a cycle of denaturation at 94°C for 2 min. Then 40 cycles of PCR amplification was carried out as follows: (denaturation) 94°C for 15 sec, (annealing) 56°C for 30 sec, (extension) 68°C for 30 sec, and final extension of 1 cycle at 68°C for 5min. For the nesting step, 35 cycles of reaction were carried out using the initial PCR amplification conditions as above.

**Overlapping PCR**

Reconstruction of *U3-gag* fragment: First, the *U3-R* and *R-gag* fragments were amplified from the respective plasmid clones generated above by PCR and then treated with T4 DNA polymerase to remove 3'-A overhangs, in case incorporated during PCR, in order to make the fragments blunt-ended. These two types of fragments were joined by the overlapping PCR method as previously performed [25] by using the high-fidelity PCR system (Roche) and the primer pairs U-F2 and R-R2 (Table S1). The amplified *U3-gag* fragments were cloned and sequence-analyzed to check for any nucleotide-errors. Using a good clone with no sequence error, the *U3-gag* fragment was reamplified and treated with T4 DNA polymerase and used in the following step.

Reconstruction of the complete 5'-half and 3'-half fragments: The 5.3kb fragments were reamplified from the chosen clones using a *gag*-specific primer (*gag*-F: TAGCGGAGGCTAGAAGGAGAGAG) and the primer 5-R2 (Table S1). These amplifications were done in 25 cycles of PCR using the Expand high fidelity PCR system (Roche). The amplified fragments were treated with T4 DNA polymerase and then mixed with *U3-gag* fragments at an equimolar ratio to combine them in the overlapping PCR method using U-F2 and 5-R2 primer pairs. The resulting amplified fragments were cloned into pCR-XL-TOPO vector and sequenced to validate the correct fusion of fragments.

Similarly, 3'-half fragments amplified from the selected plasmid clones were processed and combined with the *U3-gag* fragments in the overlapping PCR method using 3-F2 and R-R2 primer pairs. The amplified fragments were also cloned into pCR-XL-TOPO vector and the fragment junctions were sequence-checked.

**Analyses of short vRNA splice junctions**

To check whether 5'-MSD (GT-to-GC) mutation of both standard HIV_JRCSF_ and a reconstructed RV clone can affect vRNA splicing, we transfected equivalent amounts of these viral DNAs in TZM-bl cells using X-tremeGENE 9 transfection reagent (Roche, USA), and 48h later we isolated total RNA, including vRNA, from cells by using the nucleospin RNA isolation kit (TaKaRa-Clontech). DNase treatment was carried out in columns by following the manufacturer’s instruction to eliminate any contaminating DNA with the RNA preparations. RNAs were eluted with equal volumes of RNase-free water and stored at -70°C.

Viral short spliced transcripts, unspliced *gag* transcripts and β-actin RNA (as input control) were amplified by RT-PCR using the protocol as follows. The primer pairs used were:

HIV-*LTR/tat*

526-S, CAATAAAGCTTGCCTTGAGTGC

6266-AS, GCTCTCATTGCCACTGTCTTCTGCTCTTTC

HIV-*LTR/gag*

526-S, CAATAAAGCTTGCCTTGAGTGC

1322-AS, GCCTTCTGATAATGCTGAAAACA

β-actin

Sense, GCACCACACCTTCTACAATG

Antisense, TGCTTGCTGATCCACATCTG

For all RT-PCR reactions, 25µl of the reaction mixture consisted of 0.3µM of each primer, 1x reaction buffer, and 0.5µl of superscript III RT/platinum Taq HiFi (Life Technologies). For HIV-*LTR/tat* amplification, 3µl of total RNA was used as template. cDNA synthesis was performed in one cycle at 55°C for 15 min, followed by 1 cycle of denaturation at 94°C for 2 min. Then 35 cycles of PCR amplification was carried out as follows: (denaturation) 94°C for 15 sec, (annealing) 55°C for 30 sec, (extension) 68°C for 40 sec, and final extension of 1 cycle at 68°C for 5 min. For HIV-*gag* and β-actin gene amplification, 3µl and 1µl of total RNA were used as template, respectively. cDNA synthesis for both was performed at 55°C for 15 min, followed by denaturation at 94°C for 2 min. Then 35 cycles of PCR amplification was carried out as follows: (denaturation) 94°C for 15 sec, (annealing) 58°C for 30 sec, (extension) 68°C for 30 sec, and final extension of 1 cycle at 68°C for 5 min. The amplified products were cloned into pCR-XL-TOPO-TA vector (Life Techmnologies) and 4-5 clones from each were sequence-analyzed.

Figure 6D and Figure S2 shows the RT-PCR-amplified products and vRNA splice junctions, respectively.
