# Supplementary figures and images for: Prolonged persistence of a novel replication-defective HIV-1 variant in plasma of a patient on suppressive therapy
Source: Virol J. 2016 Sep 21;13:157. doi: 10.1186/s12985-016-0617-0 (PMC5031319; doi:10.1186/s12985-016-0617-0)

## Slide 1
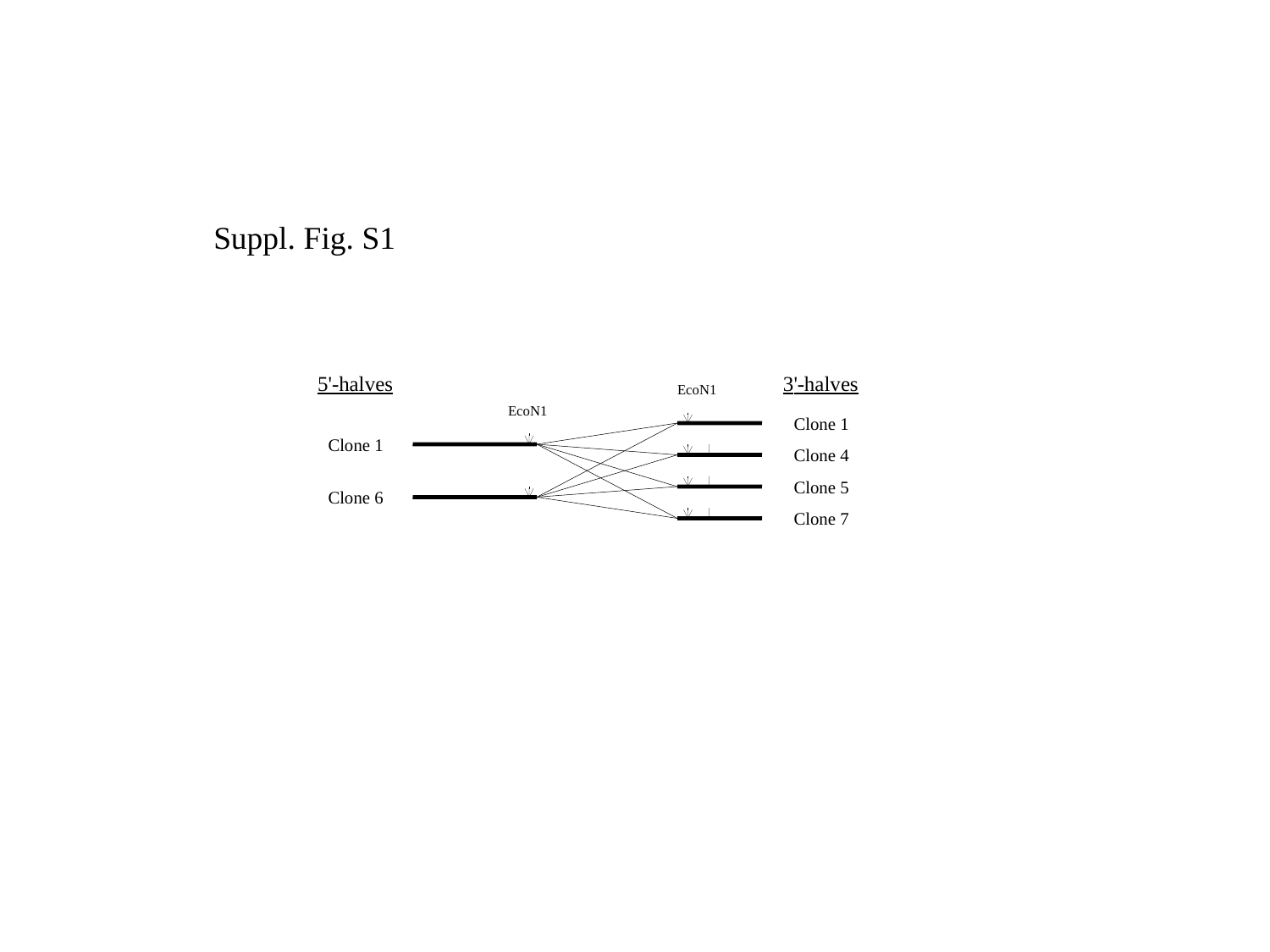

Suppl. Fig. S1
5'-halves
3'-halves
EcoN1
EcoN1
Clone 1
Clone 1
Clone 4
Clone 5
Clone 6
Clone 7

Supplement: Additional file 2: Figure S1. — Combinatorial strategy to build 8 residual vDNA clones. Each 5′-half clones (i.e., clone 1 and 6 shown on the left) were combined with four different 3′-half clones on the right through the overlapping EcoN1 sites (↓). An additional EcoN1 site (׀) present in all 3′-half clones, except clone 1 (see right), was abolished by site-directed mutagenesis prior to the combination. (PPT 55 kb) [file 12985_2016_617_MOESM2_ESM.ppt]

## Slide 1
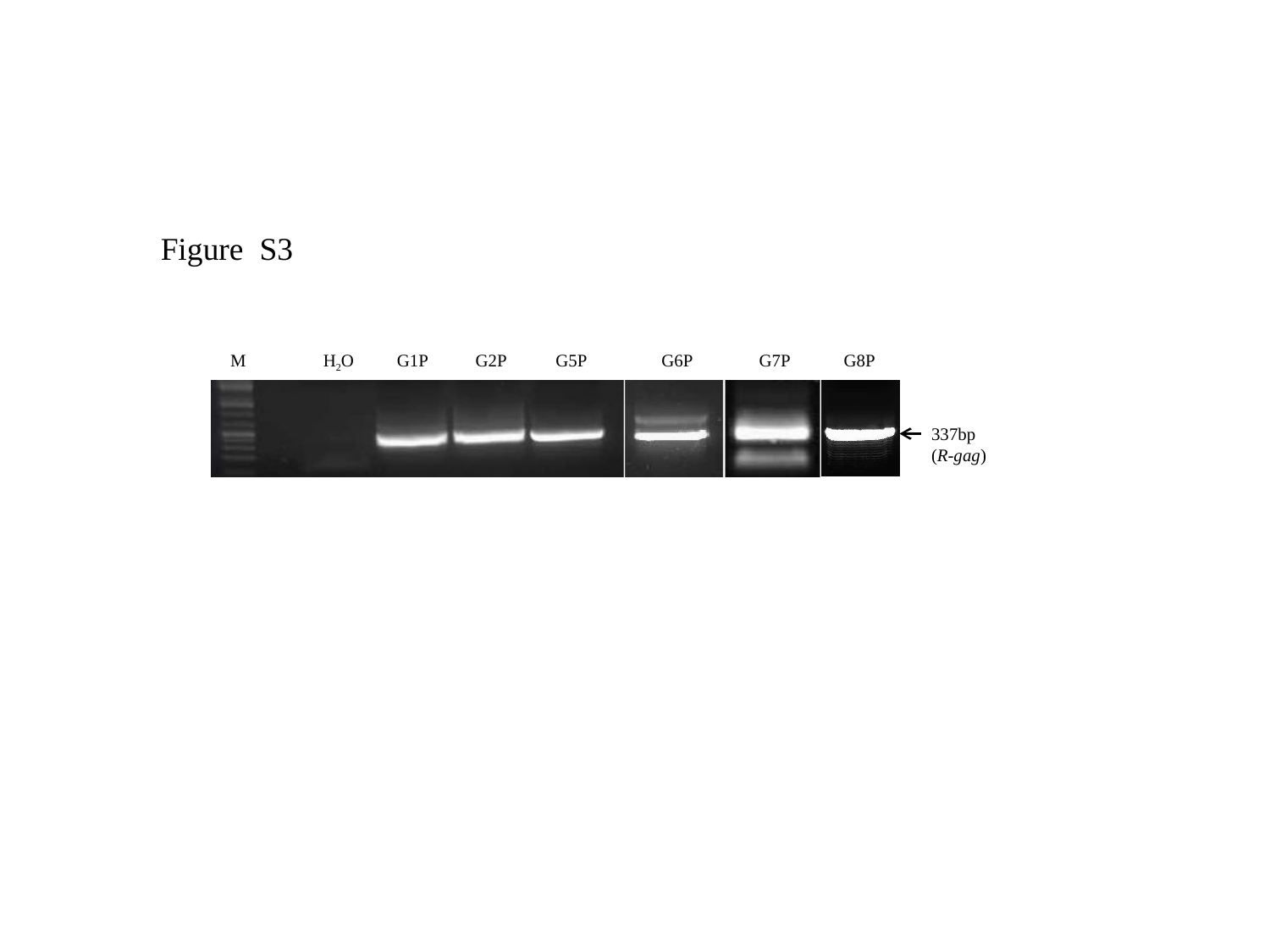

Figure S3
M
H2O
G1P
 G2P
G5P
G6P
 G7P
 G8P
337bp (R-gag)

Supplement: Additional file 4: Figure S3. — Amplification of R-gag fragments by RT-nested PCR. Longitudinally-collected vRNA samples (i.e., G1P, G2P, etc.) were used as targets for amplification. (PPT 105 kb) [file 12985_2016_617_MOESM4_ESM.ppt]
