# Supplementary material for: Prolonged persistence of a novel replication-defective HIV-1 variant in plasma of a patient on suppressive therapy
Source: Virol J. 2016 Sep 21;13:157. doi: 10.1186/s12985-016-0617-0 (PMC5031319; doi:10.1186/s12985-016-0617-0)
Supplement: Additional file 3: Figure S2. — Shift of 5′-major splice donor cleavage site toward 4nt downstream in short transcripts of HIV 5′-MSD mutants. Panel a, b, c, & d represent the group of aligned sequences derived from viral short transcripts. Top sequences in each panel represent the vDNA clones from where the transcripts were synthesized and subsequently spliced in transfected cells. HIV-JRCSF-mt and RV-1 in panel b and c represent the sequences from 5′-MSD motif mutants of HIV-JRCSF and a reconstructed RV clone, respectively; whereas RV-2a in panel d indicate the sequences of RV-1, except that the dinucleotide ‘GC’ was reverted back to the wild-type ‘GT’ form at the 5′-MSD site (underlined in red). In Panel b, one of the 5 transcripts analyzed (i.e., clone 4) showed a new splicing cleavage point located 50nt downstream of the regular 5′-MSD cleavage site. In Panel c, a viral transcript represented by clone 2 utilized the dinucleotide GC at the mutated 5′-MSD motif as a non-canonical splice donor site for RNA cleavage. (PPT 95 kb) [file 12985_2016_617_MOESM3_ESM.ppt]

## Slide 1
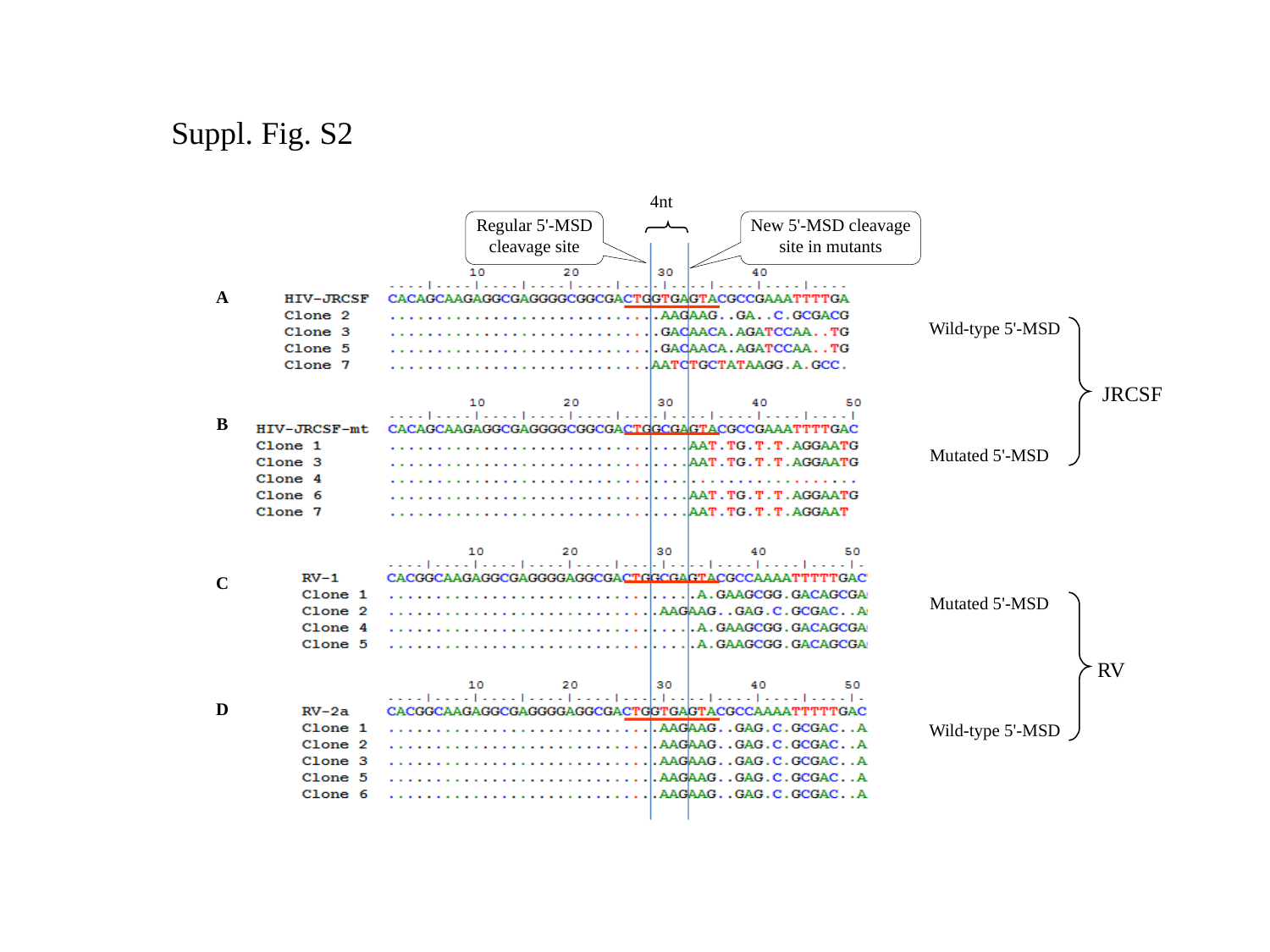

Suppl. Fig. S2
4nt
Regular 5'-MSD cleavage site
New 5'-MSD cleavage site in mutants
A
Wild-type 5'-MSD
JRCSF
B
Mutated 5'-MSD
C
Mutated 5'-MSD
RV
D
Wild-type 5'-MSD
