# Supplementary material for: Prolonged persistence of a novel replication-defective HIV-1 variant in plasma of a patient on suppressive therapy
Source: Virol J. 2016 Sep 21;13:157. doi: 10.1186/s12985-016-0617-0 (PMC5031319; doi:10.1186/s12985-016-0617-0)
Supplement: Additional file 5: Table S1. — Primers used in the RT-nested PCR for various fragment amplifications. (DOC 30 kb) [file 12985_2016_617_MOESM5_ESM.doc]

**Table S1**. Primers used in the RT-nested PCR for various fragment amplifications

| Amplified fragments | Reaction steps | Primer ID and sequence (5' to 3') |
| --- | --- | --- |
| 337bp (R-gag) | RT-PCR | (R-F1) GATCTGAGCCTGGGAGCTCTCT  (R-R1) TTCCCCCTGGCCTTAACTGAAT |
|  | Nesting | (R-F2) TAGGGAACCCACTGCTTAAGCC  (R-R2) TCCCATTTGTCCAATTCTCCCC |
| 410bp (U3-R) | RT-PCR | (U-F1) TACACGCCAGGGCCAGGGATCA  (U-R1) AAGGCAAGCTTTATTGAGGCTTA |
|  | Nesting | (U-F2) TGTGTTTTGGATGGTGCTTCAAG  (U-R2) TTTATTGAGGCTTAAGCAGTGG |
| 3.5kb | RT-PCR | (3-F1) GAGAGCAAGAAATGGAGCCAGTAGA  (3-R1) CCACATCAGCACTTCTCTCTCTGGGT |
|  | Nesting | (3-F2) CCTAGACTAGAGCCCTGGAAGCATC  (3-R2) CCATGCAGGCTTATAGGGTGTAACAAG |
| 5.3kb | RT-PCR | (5-F1) AGTAGTGTGTGCCCGTCTGTTGTGTG  (5-R1) CTGACTGTCTTGAGGAGCTCTTCGTCG |
|  | Nesting | (5-F2) TGTGTGACTCTGGTAACTAGAGATCCCTCA  (5-R2) TGTCTCCGCTTCTTCCTGCCATAGG |

Primer pairs A through H indicated in Figure 2A are as follows: A, R-F1 and R-F2; B, R-R1 and R-R2; C, U-F1 and U-F2; D, U-R1 and U-R2; E, 5-F1 and 5-F2; F, 5-R1 and 5-R2; G, 3-F1 and 3-F2; H, 3-R1 and 3-R2.
